# Signaling through the TGF Beta-Activin Receptors ALK4/5/7 Regulates Testis Formation and Male Germ Cell Development

**DOI:** 10.1371/journal.pone.0054606

**Published:** 2013-01-16

**Authors:** Denise C. Miles, Stephanie I. Wakeling, Jessica M. Stringer, Jocelyn A. van den Bergen, Dagmar Wilhelm, Andrew H. Sinclair, Patrick S. Western

**Affiliations:** 1 Centre for Reproduction and Development, Monash Institute of Medical Research, Monash University, Clayton, Victoria, Australia; 2 Department of Paediatrics, University of Melbourne, Murdoch Childrens Research Institute, Royal Children's Hospital, Parkville, Victoria, Australia; 3 Department of Anatomy and Developmental Biology, Monash University, Clayton, Victoria, Australia; Clermont Université, France

## Abstract

The developing testis provides an environment that nurtures germ cell development, ultimately ensuring spermatogenesis and fertility. Impacts on this environment are considered to underlie aberrant germ cell development and formation of germ cell tumour precursors. The signaling events involved in testis formation and male fetal germ cell development remain largely unknown. Analysis of knockout mice lacking single Tgfβ family members has indicated that Tgfβ's are not required for sex determination. However, due to functional redundancy, it is possible that additional functions for these ligands in gonad development remain to be discovered. Using FACS purified gonadal cells, in this study we show that the genes encoding Activin's, TGFβ's, Nodal and their respective receptors, are expressed in sex and cell type specific patterns suggesting particular roles in testis and germ cell development. Inhibition of signaling through the receptors ALK4, ALK5 and ALK7, and ALK5 alone, demonstrated that TGFβ signaling is required for testis cord formation during the critical testis-determining period. We also show that signaling through the Activin/NODAL receptors, ALK4 and ALK7 is required for promoting differentiation of male germ cells and their entry into mitotic arrest. Finally, our data demonstrate that Nodal is specifically expressed in male germ cells and expression of the key pluripotency gene, Nanog was significantly reduced when signaling through ALK4/5/7 was blocked. Our strategy of inhibiting multiple Activin/NODAL/TGFβ receptors reduces the functional redundancy between these signaling pathways, thereby revealing new and essential roles for TGFβ and Activin signaling during testis formation and male germ cell development.

## Introduction

Spermatogenesis and oogenesis are founded on the development of the male and female germ cell lineages in the fetal testis and ovary, respectively. In mice, primordial germ cells populate the developing gonads at approximately embryonic day (E)10.5 and differentiate down the spermatogenic or oogenic pathways in response to their respective environments [1], [2], [3], [4]. However, the molecular pathways directing male and female germ line development are poorly understood, even though these processes are crucial for later fertility and for preventing germ cell tumours.

Testis development is initiated in the XY bipotential gonad through expression of Sry (Sex determining region of Chromosome Y), which initiates expression of several testis specific genes including Sox9 (Sry-box containing gene 9) and Amh (anti-Mullerian hormone) [5]. SOX9 promotes differentiation of the supporting cells into Sertoli cells, which proliferate and form cords in response to ligands such as FGF9 (fibroblast growth factor 9) [6], [7], [8]. The testis cords enclose the germ cells and define the interstitial space, where Leydig cells differentiate and reside. Sry is normally absent in XX females, allowing pathways driven by Wnt4 (wingless-related MMTV integration site 4) Rspo1 (R-spondin homolog), Ctnnb1 (β catenin) and Foxl2 to promote ovarian development [8], [9], [10], [11], [12], [13], [14], [15].

At the onset of testis or ovary development germ cells enter the male or female developmental pathways in response to signaling pathways that are largely unknown [1], [3], [4], [16]. The earliest indication of sex-specific germ cell development is their entry into mitotic arrest in a testis or meiosis in an ovary, which occurs from E12.5 and E13.5, respectively [3]. Entry of germ cells into meiosis requires the retinoic acid (RA) responsive gene, stimulated by retinoic acid gene 8 (Stra8), for which RA supplied by the adjacent mesonephros is considered to be an activator [17], [18], [19]. In testes, RA activity is blocked by the RA metabolising enzyme encoded by cytochrome P450, family 26, subfamily b, polypeptide 1 (Cyp26B1) [17]. These mechanisms may also be impacted by fibroblast growth factor (FGF) signaling, which is required for testis development and male germ cell survival, to repress Stra8, and to promote male germ line development [20], [21], [22], [23], [24]. Recent findings suggest that additional pathways are also involved [2], [25], [26], but the signaling processes directing male germ cell development are still poorly understood.

The earliest sign of male germ cell development is the initiation of mitotic arrest, which occurs between E12.5 and E14.5 and involves activation of a number of G1-S phase check-point controlling proteins, including Retinoblastoma and p27^KIP1^ [27], [28], [29]. Male germ cell differentiation also involves upregulation of male germ line markers such as DPPA4, DNMT3L and PIWIL2 [30], [31], post transcriptional repression of OCT4 and transcriptional repression of Sox2 and Nanog, which are maintained in germ cell derived teratomas [30], [32], [33], [34], [35].

The transforming growth factor (TGF) super family contains more than 40 ligands, including TGFβ1−3, Activin A and B and NODAL, which initiate signaling in many cellular contexts. TGFβ proteins form homo- or hetero-dimers and bind their specific type II receptor, before forming a complex with their respective type I receptors, and activating signal transduction [36], [37]. Receptor activation results in phosphorylation of effector proteins of the mothers against decapentaplegic (SMAD) family, which are transported to the nucleus, where they activate transcription of target genes. The identity of the receptors and SMADs activated by each TGF ligand differs, providing specificity of action. However, since target overlap exists between both the receptors and activated SMADs, there is considerable potential for functional redundancy between different TGFβ mediated signaling pathways.

After sex determination the Tgfβ family members Inhba and Inhbb are expressed at higher levels in the somatic cells of the developing testis than the somatic cells of the ovary [36], [38]. Inhba, expressed by Leydig cells, is required for proper Sertoli cell proliferation and testis cord expansion from E15.5 [39]. Loss of function mutations in Inhba results in increased germ cell numbers during the later stages of fetal testis development [40]. Mice that have ActivinβA (encoded by Inhba) replaced with the less bioactive ActivinβB (encoded by Inhbb) evade Inhba −/− lethality and display delayed spermatogenesis [41], [42]. Inhbb is required for proper formation of the coelomic vessel in the testis [43], but additional roles in testis determination and germ cell development remain unknown. Similarly, the impact of compound loss of function mutations in Inhba and Inhbb on testis and germ cell development remain unknown.

Tgfb1-Tgfb3 are expressed in the embryonic testis, with Tgfb1 expression detected in Sertoli cells, Tgfb2 in Sertoli cells and Leydig cells and Tgfb3 in peritubular myoid cells and germ cells [44], [45]. Loss of function analyses in mice indicate that individual TGFβs are not required for somatic cell sex determination [44]. However, compensation between TGFβ ligands may obscure unknown functions in testis and germ cell development. Supporting this, loss of the TGFβ co-receptor, Tgfbr3, (also known as betaglycan) results in reduced fetal Leydig cell function and compromised testis cord structure, indicating that TGFβ signaling is required for testis development [46].

In this study, using ALK5 and ALK4/ALK5/ALK7 inhibitors in ex vivo gonad cultures we demonstrate that loss of TGFβ function depleted SMAD phosphorylation, resulting in almost complete disorganization of the developing testis cords in embryonic XY gonads, and errant entry of germ cells into meiosis. We also demonstrate that blocking Activin-TGFβ signaling in E12.5 testes significantly reduces Sertoli cell proliferation from E12.5 and prevents entry of male germ cells into mitotic arrest. Finally, we showed that Nodal was highly, and specifically, expressed by male germ cells and that blocking signaling through ALK4/5/7 led to reduced Nanog expression, indicating that NODAL regulates germ cell pluripotency through Nanog. Combined, these data reveal new and important roles for Activin-TGFβ signaling in formation of the embryonic testis and regulation of male germ cell development.

## Results

**Figure 1:**
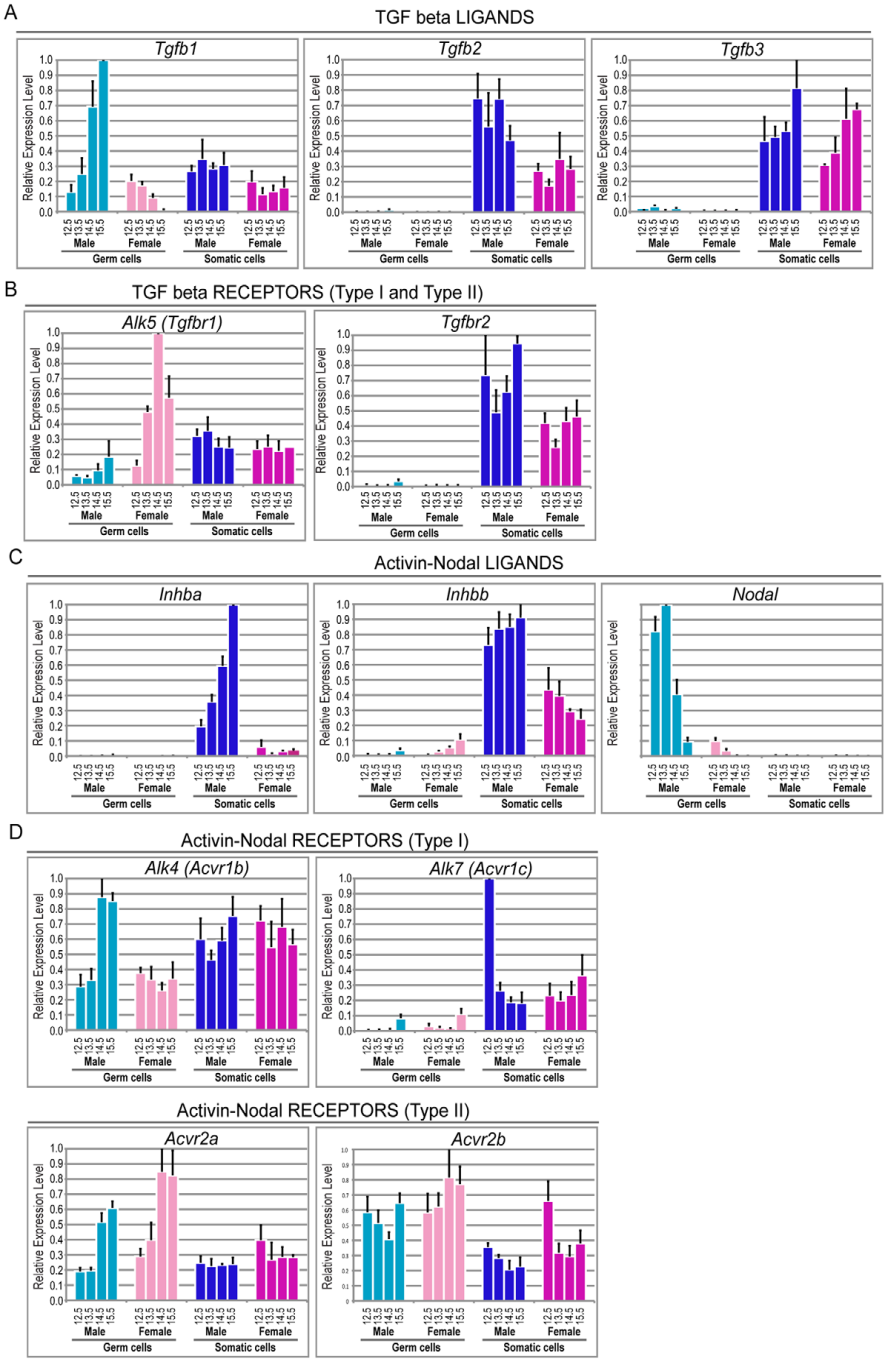
Inhba, Inhbb, Tgfb2 and Tgfb3 are expressed in the somatic cells, whilst Nodal is expressed in the germ cells of the developing gonad. Quantitative real time PCR analysis of: A) ligands Tgfb1, Tgfb2, Tgfb3; B) type I and II Tgf receptors Alk5 and Tgfbr2; C) ligands Inhba, Inhbb and Nodal; D) Activin/Nodal type I receptors Alk4, Alk7, and type II receptors Acvr2a, Acvr2b in FACS purified E12.5–E15.5 XY male germ cells (light blue) and XX female germ cells (light pink) male somatic cells (dark blue) and female somatic cells (dark pink). Expression was normalized to Mapk1 and Canx. Error bars represent standard error of three biological replicates.

We first used qRT-PCR to assess the expression of Inhba, Inhbb, Tgfb1-3 and Nodal and their respective type I (Alk4, Alk5 and Alk7) and type II (Acrv2a and Acrv2b) receptors in FACS purified XX female and XY male E12.5–E15.5 somatic and germ cells (Figure 1). The observed expression patterns are summarized as follows.

### Expression of Tgfb's and their receptors indicate a role in somatic cell development during testis formation

Of Tgfb1-3, Tgfb1 was the only gene expressed at appreciable levels in germ cells (Figure 1A). Initially, Tgfb1 levels were relatively low in male and female germ cells. However, during E13.5–E15.5 Tgfb1 expression increased 8 fold in male germ cells (p<0.05, E12.5 vs E15.5), and decreased 7 fold between E14.5 and E15.5 in female germ cells (p<0.05, E12.5 vs E15.5). Tgfb1 was also expressed in male and female somatic cells, but at similar, relatively constant levels in both sexes and at all stages. Like Tgfb1, Tgfb2 and Tgfb3 were expressed in male and female somatic cells. However, in germ cells expression of Tgfb2 and Tgfb3 was negligible. The TGFβ type I receptor, Alk5, was expressed at its highest levels in female germ cells. Alk5 expression was very low in male germ cells, but it was expressed at similar, moderate levels in male and female germ cells (Figure 1B). The type II receptor Tgfbr2 was expressed at high levels in male and female somatic cells, but was essentially undetectable in male and female germ cells (Figure 1B).

### Activins A and B are expressed by male somatic cells, while Nodal is expressed by male germ cells

At E12.5, Inhba (which encodes Activin A) was expressed at three fold higher levels in male somatic cells compared to female somatic cells, and by E15.5 Inhba levels had increased to levels 28 fold higher in male somatic cells than those observed in female somatic cells (p<0.05, E12.5 male vs female somatic cells). At E12.5 Inhbb (which encodes ACTIVIN B) was expressed at similar levels in male and female somatic cells, but by E15.5 Inhbb expression in female somatic cells had decreased to levels four fold lower than those observed in male somatic cells (p<0.05, E15.5 male germ vs female somatic cells). Expression of Inhba and Inhbb was essentially undetectable in germ cells (Figure 1C). By contrast, Nodal was expressed at high levels only in E12.5–E15.5 germ cells. Nodal expression was 8–10 fold higher in E12.5 and E13.5 male than in E12.5 and E13.5 female germ cells, respectively (p<0.05, E12.5 male germ vs female germ cells), but decreased more than 10 fold in male germ cells between E13.5 and E15.5 (Figure 1C).

### Activin/NODAL receptors are expressed by somatic and germ cells

The type I receptors for Activin and NODAL signaling are ALK4 and ALK7. Alk4 was expressed at similar levels in male and female somatic and germ cells. Although Alk4 levels were approximately two fold higher in somatic than germ cells, its expression increased two fold in male germ cells between E13.5 and E14.5 (Figure 1D). Alk7 was expressed at higher levels in male and female somatic cells compared to germ cells. Expression of Alk7 was negligible in E12.5–E14.5 germ cells. The type II receptors Acrv2a and Acvr2b were also expressed at similar levels in all cell types, although there was a moderate increase in Acrv2a expression in E14.5–E15.5 male and female germ cells, and Acvr2b levels were moderately higher in germ cells than somatic cells (Figure 1D).

### Inhibition of ALK4/5/7 or ALK5 at E11.5 prevents testis cord formation

**Figure 2:**
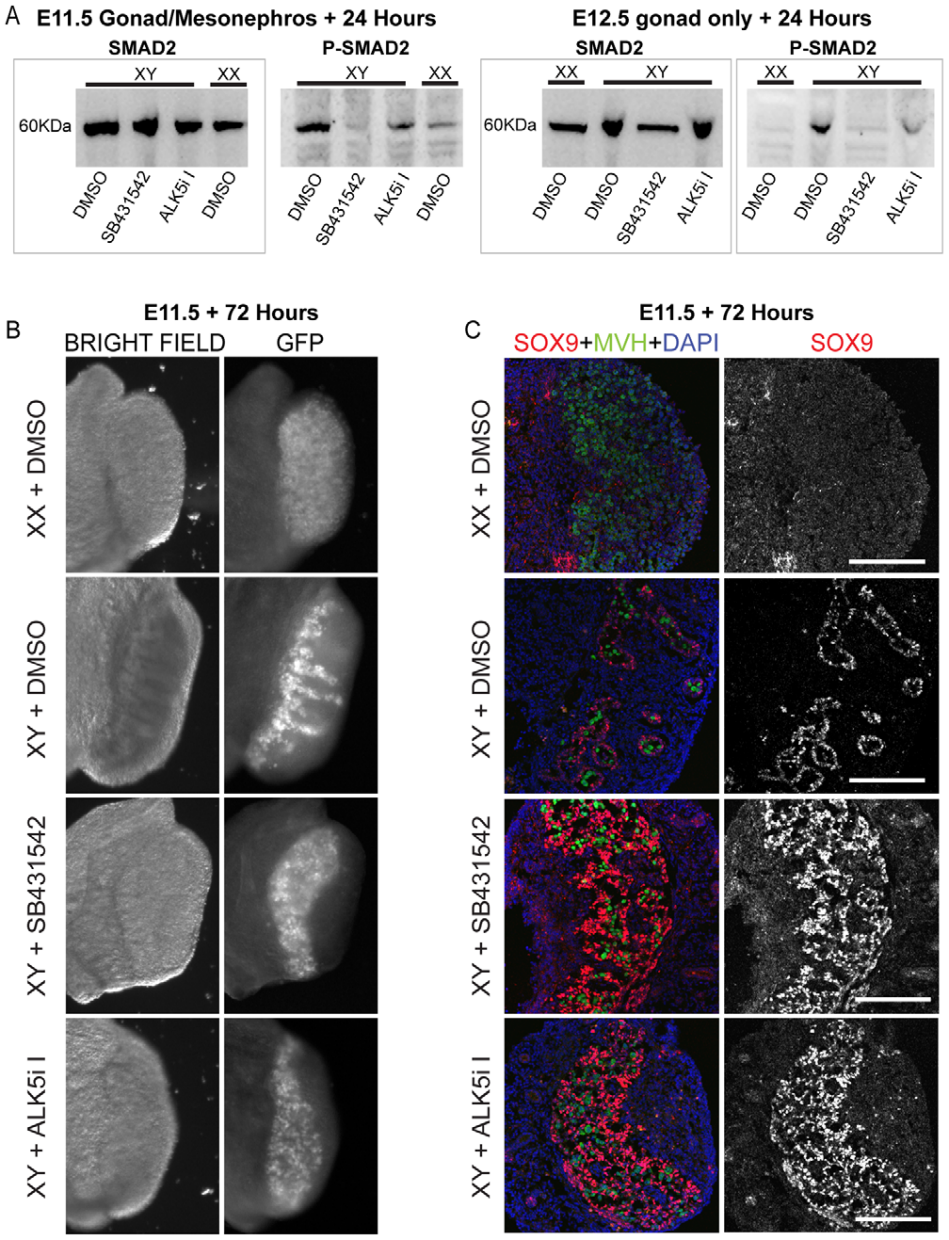
E11.5 XY gonads cultured in the presence of ALK4/5/7 inhibitor (SB431542) or ALK5 inhibitor (ALK5i-I) exhibit disrupted testis cord formation. A) E11.5 (gonad/mesonephos) and E12.5 (gonad only) XY and XX gonad samples cultured for 24 hours in the presence of DMSO, SB431542 or ALK5i-I and analysed using immunoblotting with antibodies specific for SMAD2 and phosphorylated SMAD2 (P-SMAD2). B) Bright field and GFP images of XY and XX gonads collected from E11.5 Oct4-GFP transgenic embryos and cultured for 72 hours in the presence of DMSO, SB431542 or ALK5i-I. C) Immunofluorescence analysis of E11.5 XY and XX gonads cultured for 72 hours in the presence of DMSO, SB431542 or ALK5i-I. SOX9 staining is in red, while MVH (green) staining marks the germ cells. DAPI (blue) marks the nucleus. Scale bars; 200 μm.

To elucidate the role of Activin, TGFβ and NODAL signaling in testis development we cultured male and female E11.5 gonad/mesonephros and E12.5 gonad only samples ex vivo with a vehicle control (DMSO), an ALK4/5/7 inhibitor (SB431542), or an ALK5 inhibitor (ALK5i-I) [47], [48], [49], [50], [51], [52], [53], [54], [55]. To confirm blockage of signaling through ALK4/5/7 and ALK5 we used immuno-blotting to assess total SMAD2 and phosphorylated SMAD2 (P-SMAD2) levels in samples that were treated for 24 hours (Figure 2A). All (XY and XX) E11.5 gonad/mesonephros samples and the E12.5 gonad only samples (DMSO, SB431542 and ALK5i-I treated) contained substantial levels of SMAD2 protein. However, in both the E11.5 and E12.5 samples the levels of P-SMAD2 were substantially higher in male samples than in females. P-SMAD2 was detected at low levels in the female E11.5 gonad/mesonephros samples, but was not detected in the E12.5 ovaries. Significantly, treatment of the male samples with SB431542 reduced P-SMAD2 levels to almost undetectable levels, while ALK5i-I reduced P-SMAD2 to intermediate levels, compared to the DMSO treated controls (Figure 2A).

To determine the effect of blocking signaling through ALK4/5/7 and ALK5 on gonad development we next cultured E11.5 XY and XX gonad/mesonephros samples with DMSO, SB431542 or ALK5i-I for 72 hours (approximately equivalent to E14.5). We used Oct4-GFP transgenic embryos for this analysis, allowing the visualization of germ cells in the cultured gonad samples. Expression of GFP in these embryos revealed the position of the germ cells, providing gross structural information as well as relative germ cell survival in the developing gonad. E11.5 XY gonads cultured in the presence of DMSO formed testis cords, which were evident through bright field and fluorescence examination (Figure 2B). Visualisation of GFP demonstrated that the germ cells resided within well-developed cords (Figure 2B). XX gonads cultured in the presence of DMSO, SB431542 and ALK5i-I had typically ovarian morphology, with germ cells spread throughout the gonad (Figure 2B). No differences were observed between the inhibitor treated and untreated female gonads. However, when XY gonads were cultured in the presence of SB431542 or ALK5i-I, testis cord formation was disrupted and the germ cells were spread throughout the gonad (Figure 2B).

### Inhibition of ALK4/5/7 or ALK5 disrupts testis cord organisation and localisation of SOX9 expressing cells

Morphological analysis indicated that treatment of E11.5 XY gonads with SB431542 or ALK5i-I might lead to sex reversal. Therefore, we used immunofluorescence to further analyse structural and molecular characteristics in these gonads. SOX9 is a marker of early Sertoli cell development in XY gonads, while MVH (mouse vasa homologue) is a marker of male and female germ cells. As expected, SOX9 was not detected in DMSO treated XX gonads and MVH expressing germ cells were observed throughout the gonad. XY gonads treated with DMSO expressed SOX9 in Sertoli cells, which outlined well-developed cords that contained MVH expressing germ cells (Figure 2C). In contrast, while both SB431542 and ALK5i-I treated XY gonads contained SOX9 positive cells, these cells were distributed throughout the gonad and did not outline well-formed testis cords. Consistent with this lack of testis cord structure, germ cells were localised throughout the SB431542 and ALK5i-I treated XY gonads in a distribution similar to that observed in DMSO treated XX gonads. Despite the lack of testis cords, the ovarian somatic cell marker FOXL2 was not detected in XY gonads treated with SB431542 or ALK5i-I for 72 hours, indicating that the somatic cells of these gonads were not sex reversed.

### Signaling through ALK4/7 is required for Sertoli cell proliferation

**Figure 3:**
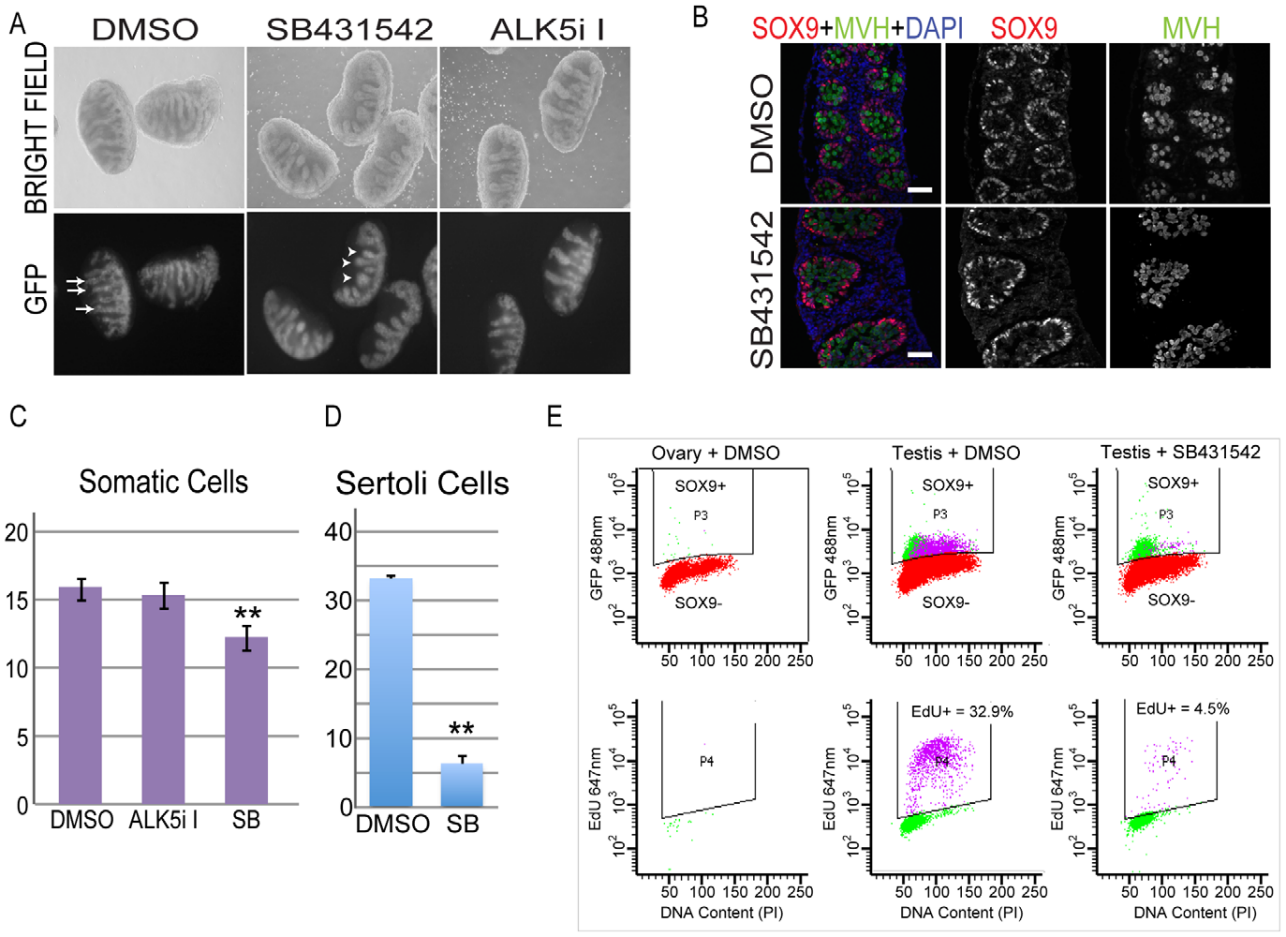
Inhibition of ALK4/5/7 decreases Sertoli cell proliferation in E12.5 fetal testes. A) Bright field and GFP images of male testes collected from E12.5 Oct4-GFP transgenic embryos and cultured in the presence of DMSO, SB431542 or ALK5i-I for 72 hours. Arrows indicate well-developed testis cords marked by GFP positive germ cells in DMSO treated controls. Arrow-heads indicate stunted testis cords marked by GFP positive germ cells in SB431542 treated testes. B) Immunofluorescence analysis of the same testes using SOX9 and MVH antibodies. SOX9 staining is in red, while MVH (green) staining marks the germ cells. DAPI (blue) is included as a nuclear marker. Scale bars; 50 μm. C–D) Flow cytometric analysis of somatic cell (MVH negative cells, Figure 5a) and Sertoli cell (SOX9 positive cells) proliferation based on EdU incorporation of E12.5 testes cultured for 72 hours with DMSO (n = 10, MVH negative cells; n = 3 SOX9 positive cells), ALK5i-I (n = 3) or SB431542 (n = 10, MVH negative cells; n = 3 SOX9 positive cells). In C and D the error bars represent standard error. ** indicates significant difference compared to DMSO (p<0.01). E) Typical example of a flow cytometric analysis of Sertoli cells in E12.5 testes cultured for 72 hours with DMSO or SB431542. SOX9 positive germ cells were gated from somatic cells (top panels) and analysed for active proliferation by measuring incorporation of EdU during S-phase (bottom panels).

To determine whether the inhibition of signaling through ALK4/5/7 also disrupted cord structure after testis cords had formed, we treated E12.5 testes with SB431542 and ALK5i-I. Although robust testis cords were evident within E12.5 testes treated with DMSO, SB431542 and ALK5i-I for 72 hours, cord growth was affected in samples treated with SB431542 and ALK5i-I, with cords exhibiting a stunted and wider appearance compared to DMSO treated gonads (Figure 3A). This phenotype was highly reproducible, occurring in all experiments performed (>10), and was used as a routine marker for these gonads in culture. However, based on whole mount examination of GFP, all germ cells were enclosed in the testis cords. Moreover, immunofluorescent analysis of SOX9 demonstrated that Sertoli cells clearly delineated the cords in these testes (Figure 3B). Consistent with this, the ovarian marker FOXL2 was not detected. To determine the basis of this stunted cord phenotype we examined proliferation of the somatic cells in these gonads. We routinely use MVH to separate germ cells from somatic cells in a flow cytometric assay that allows assessment of the proportion of cells actively engaged in S-phase (by measuring EdU incorporation) and cell cycle state by cellular DNA content (PI staining) [27], [28], [56]. Flow cytometric analysis of the MVH negative cells, which consist of a range of cell types including Sertoli and interstitial cells, in E12.5 testes cultured for 72 hours revealed significantly lower numbers of EdU positive (12.3±0.81%; SEM, n = 10) somatic cells in SB431542 treated testes compared to ALK5i-I (15.3±0.9%; SEM, n = 3), or DMSO treated testes (15.9% ±0.6; SEM, n = 10) (p = 0.001) (Figure 3C). To determine which somatic cell type was affected in the SB431542 treated testes we performed flow cytometry using SOX9 to separate the Sertoli cells from germ and interstitial cells (Figure 3D). This analysis revealed that 6.3%±1.1 (SEM, n = 3) of Sertoli cells incorporated EdU in SB431542 treated testes compared to 33.2%±0.4 (SEM, n = 3) in DMSO treated controls (Figure 3E), clearly demonstrating a significant reduction in Sertoli cell proliferation as a result of abrogated ALK4/5/7 signaling (p<0.001) (Figure 3E). Moreover, in E12.5 fetal testes treated with SB431542 or DMSO for 24 hours, 12.6% and 20.8% of Sertoli cells were detected in S-phase, respectively, demonstrating that blocking signaling through ALK4/5/7 resulted in reduced Sertoli cell proliferation very early in testis development.

### Germ cells enter meiosis in E11.5 XY gonads treated with SB431542 or ALK5i-I

**Figure 4:**
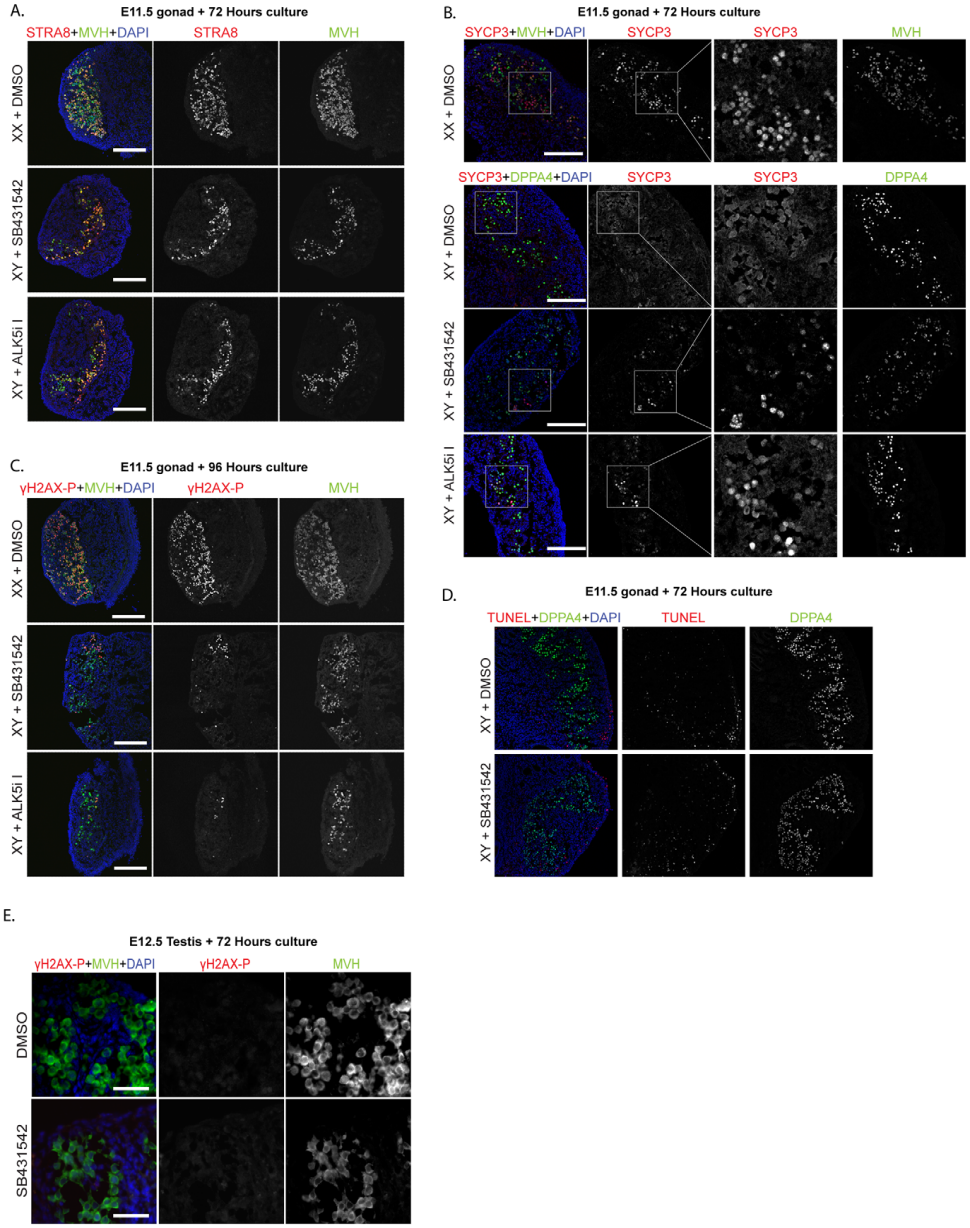
Blocking ALK4/5/7 or ALK5 in E11.5 XY gonads, but not E12.5 testes, permits germ cell entry into meiosis. Immunofluorescence analysis of: A–C) E11.5 XY and XX gonads cultured for 72 hours or 96 hours in the presence of DMSO, SB431542 or ALK5i-I. STRA8 (A), SYCP3 (B) and γH2AX-P (C) staining is in red, while MVH or DPPA4 (green) staining marks the germ cells. DAPI (blue) is included as a nuclear marker. Scale bars; 200 μm. D) TUNEL staining of E11.5 gonads cultured for 72 hours in the presence of DMSO or SB431542. Red staining marks TUNEL positive cells, while germ cells are marked by DPPA4 staining in green. DAPI (blue) is included as a nuclear marker. Scale bars; 200 μm. **E**) E12.5 testes cultured for 72 hours in the presence of DMSO or SB431542. γH2AX-P staining is in red, while MVH (green) staining marks the germ cells. DAPI (blue) is included as a nuclear marker. Scale bars 50 μm.

Male germ cell sex determination is dependent on signaling from testicular somatic cells, and failure of this mechanism blocks male germ cell differentiation and allows germ cells to enter meiosis [3], [4]. Since E11.5 XY gonads treated with SB431542 or ALK5i-I were highly disorganized and germ cells were not contained within testis cords, we speculated that this might affect male germ cell development, and allow entry into meiosis, an obvious ovarian characteristic at this stage. Phosphorylated γH2AX (γH2AX-P), synaptonemal complex protein 3 (SYCP3) and STRA8 mark female germ cells entering meiosis. As expected, STRA8, SYCP3 and γH2AX-P were robustly expressed in the germ cell population of DMSO treated XX gonads. With the exception of a small number of STRA8 positive germ cells close to the mesonephric-gonadal border, these proteins were absent in germ cells of DMSO treated XY gonads that had been cultured for 72 (STRA8, SYCP3) or 96 hours (γH2A-X) (Figure 4A–C). In contrast to XY control treated gonads, essentially all germ cells were positive for STRA8 in E11.5 XY gonads treated with SB431542 or ALK5i-I (Figure 4A). However, a smaller proportion of the germ cells in XY SB431542 or ALK5i-I gonads were SYCP3 or γH2AX-P positive than were STRA8 positive (Figure 4B–C). Combined these results indicate that although blocking signaling through ALK5 alone in E11.5 XY testes was sufficient to permit most germ cells to initiate the meiotic program, only a subset of these germ cells proceeded further through the meiotic program. To determine whether germ cells entering the meiotic program under these conditions were apoptotic we performed TUNEL staining and immunofluorescence for cleaved-PARP on sections of XY gonads treated with DMSO or SB431542 for 72 hours. Although some TUNEL positive somatic cells were detected in both the DMSO and SB431542 treated gonads, their numbers were similar in both treatments and very few germ cells were TUNEL positive in either treatment (Figure 4D). Similarly, no cleaved-PARP positive germ cells were detected in either treatment (not shown).

To establish the critical timing of TGFβ function in relation to germ cell meiosis we cultured E12.5 male gonads without mesonephros for 72 hours (approximately equivalent to E15.5) and performed immunohistochemistry for STRA8, γH2AX-P and another marker of meiotic female germ cells, phosphorylated CHEK2 (P-CHEK2) [18], [56], [57]. With the exception of one sample, in which we detected five germ cells expressing γH2AX-P, STRA8, γH2AX-P and P-CHEK2 were not detected in germ cells of E12.5 testes treated for 72 hours with SB431542 or DMSO (Figure 4E, γH2AX shown), indicating that blocking TGFβ function in the testis from E12.5 does not permit germ cell entry into meiosis. As with E11.5 gonads treated with DMSO or SB431542, levels of apoptosis as assessed by TUNEL staining were low and no difference was observed between the control and treated samples (not shown).

### Signaling through ALK4/7 is required for male germ cell development and mitotic arrest

**Figure 5:**
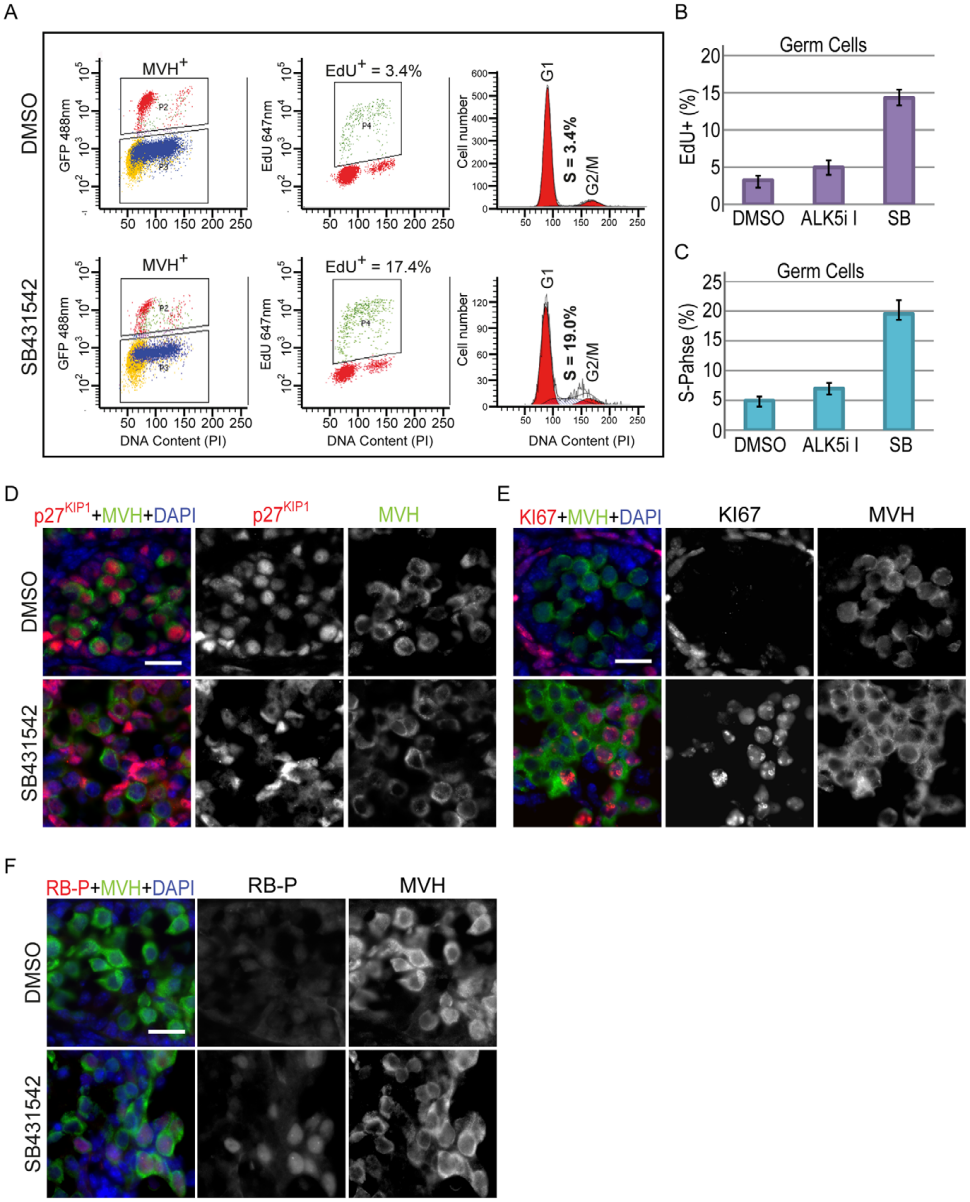
Inhibition of ALK4/5/7 disrupts germ cell cycle arrest in E12.5 testes. A) Typical example of a flow cytometric analysis of germ cells from E12.5 testes cultured for 72 hours with DMSO or SB431542. MVH positive germ cells were gated from somatic cells (left panels) and analysed for active proliferation by measuring incorporation of EdU during S-phase (middle panels) and for cell cycle stage by measuring DNA content (right panels). B–C) E12.5 testes treated with DMSO (n = 10), ALK5i-I (n = 3) or SB431542 (n = 10) for 72 hours B) Germ cell proliferation based on EdU incorporation C) ModFit analysis of germ cell cycle state, based on propidium iodide staining for DNA content. Error bars represent standard error. D–F) Immunofluorescence analysis of E12.5 testes cultured for 72 hours in the presence of DMSO or the ALK4/5/7 inhibitor SB431542. D) p27^KIP1^ (red); E. KI67 (red); F) phosphorylated RB (RB-P) (red). In D–F MVH staining (green) marks the germ cells while DAPI (blue) marks nuclear DNA. Scale bars; 20 μm.

To further examine the differentiation state of germ cells in testes treated with SB431542, ALK5i-I or DMSO, we analysed germ cell entry into mitotic arrest using an MVH antibody and flow cytometry to separate germ cells from somatic cells [27], [28] (Figure 5A). Analysis of germ cell proliferation in testes isolated at E12.5 and treated for 72 hours with DMSO, SB431542 and ALK5i-I revealed that 3.2±0.6% (SEM, n = 10 cultures), 14.3±3.3 (SEM, n = 10), 4.9±0.9% (SEM, n = 3) of the germ cell population was actively engaged in S-phase, respectively, as assessed by their incorporation of EdU (Figure 5B). Assessment of the proportion of germ cells in S-phase based on cellular DNA content corroborated these data (Figure 5B). Thus treatment with SB431542, which blocks signaling through ALK4/5/7, significantly reduced the efficiency of germ cell mitotic arrest (p<0.001) compared to DMSO, while treatment with the ALK5 specific inhibitor did not block mitotic arrest (p = 0.20) (Figure 5B).

To determine whether key markers of germ cell entry into mitotic arrest were affected in the presence of SB431542 we treated gonads in organ culture for 72 hours and performed immunofluorescent analysis of p27^KIP1^, phosphorylated RB (RB-P) and KI67 [27], [28]. Co-staining of sections for p27^KIP1^ and an antibody for the germ cell marker MVH revealed strong expression of p27^KIP1^ in the nucleus of germ cells treated with DMSO. In contrast, clusters of germ cells that did not express p27^KIP1^ were detected in gonads treated with SB431542 (Figure 5D). KI67 expression is eliminated from germ cells that have entered mitotic arrest [27], [28], [56], [58]. Analysis of gonads treated with SB431542 revealed clusters of germ cells expressing nuclear KI67, indicating that they had not entered mitotic arrest. In contrast, in DMSO treated testes none of the germ cells expressed KI67 (Figure 5E). Consistent with this, RB-P was readily detected in the nucleus of germ cells treated with SB431542, but rarely detected in germ cells of gonads treated with DMSO (Figure 5F). Combined, these results clearly demonstrated that the molecular control of G0 arrest in male germ cells was disrupted in fetal testes treated with the ALK4/5/7 inhibitor SB431452.

**Figure 6:**
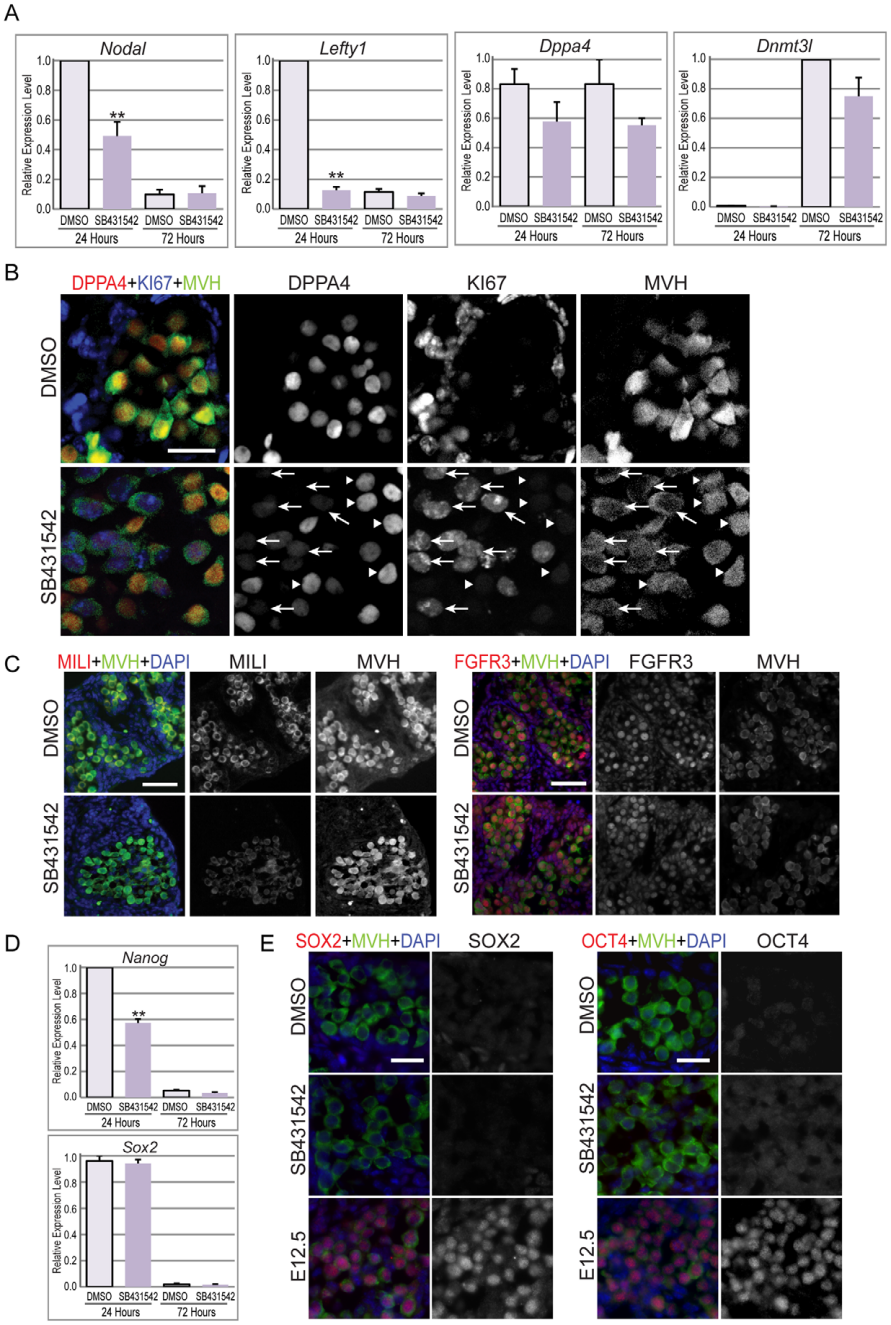
Inhibition of ALK4/5/7 inhibits male fetal germ cell differentiation. A) qRTPCR analysis of Nodal and Lefty1, and the male germ cell differentiation markers Dppa4 and Dnmt3l in E12.5 testes cultured for 72 hours with DMSO or SB431542. All of these genes are specifically expressed by the germ cells. Relative expression (± SEM, n = 3) was calculated after normalization to Mapk1 and Canx [64]. B–C) Immunofluorescence analysis of E12.5 testes cultured for 72 hours in the presence of DMSO or SB431542. B) DPPA4 is in red, KI67 is in Pacific Blue and MVH is in green. In the SB431542 treated images the arrows indicate germ cells that express low levels of DPPA4 and remain proliferative (KI67 positive), while the arrow-heads indicate germ cells that express high levels of DPPA4 and have exited the cell cycle (KI67 negative); C) MILI (red, left panel); FGFR3 (red right panel); In C–D MVH staining (green) marks the germ cells while DAPI (blue) marks nuclear DNA. Scale bars; 20 μm. D) qRTPCR analysis of Nodal and the pluripotency markers Nanog and Sox2 in E12.5 testes cultured for 72 hours with DMSO or SB431542. Relative expression (± SEM, n = 3) was calculated after normalization to Mapk1 and Canx. E) Immunofluorescence analysis of E12.5 testes cultured for 72 hours in the presence of DMSO or SB431542. SOX2 (red, left panel); OCT4 (red right panel). MVH staining (green) marks the germ cells. DAPI (blue) marks nuclear DNA. Scale bars; 20 μm.

We next assessed male germ cell differentiation by examining expression of Nodal, Lefty1, Dppa4, Dnmt3l and Mili (also known as Piwil1), which are all normally upregulated in differentiating male germ cells [28], [30], [59]. To determine whether blocking ALK4/5/7 would disrupt male specific expression of Nodal, Lefty1, Dppa4, Dnmt3l and Mili, we performed qRTPCR on E12.5 gonads treated with DMSO or SB431542. After 24 hours of culture expression levels of both Nodal and Lefty1 were significantly decreased in SB431542 treated gonads, compared to the DMSO control (p<0.05) (Figure 6A). After 72 hours of culture, Nodal and Lefty1 levels were reduced to only 10% of their peak levels in both the DMSO control and SB431542 treated gonads. Transcription of Dppa4 was not significantly reduced in SB431542 treated gonads at either 24, or 72 hours (p = 0.18), compared to the DMSO treated testes. Similarly, transcription of the male germ cell differentiation marker Dnmt3l was also similar in testes treated with the SB431542 for 72 hours (p = 0.12). It is likely that expression changes assessed by qRTPCR are underestimated due to the mix of affected (proliferating) and unaffected (arrested) germ cells in this system. To circumvent this problem and to support the qRTPCR analyses we used immunofluorescence to monitor protein levels of DPPA4, MILI, FGFR3 and KI67 on an individual germ cell basis in XY gonads isolated at E12.5 and treated for 72 hours with SB431542. Within testes treated with SB431542, DPPA4 expression was substantially reduced in a subset of germ cells compared to the DMSO control levels, whereas in the remainder of the germ cells DPPA4 levels were normal. To determine whether germ cells that lacked high levels of DPPA4 expression also escaped mitotic arrest, we co-stained sections of SB431542 treated gonads with DPPA4, KI67 and MVH (Figure 6B). Proliferative germ cells were identified by their continued expression of KI67, as loss of this marker is an accurate indicator of mitotic arrest [27], [28]. In SB431542 treated gonads, KI67 positive cells displayed very low levels of DPPA4, while KI67 negative germ cells contained high levels of DPPA4 (Figure 6B). In DMSO treated gonads all cells were KI67 negative and contained high levels of DPPA4 (Figure 6B). MILI protein expression is induced in differentiating male germ cells between E14.5 and E15.5 [28]. In two out of three samples tested, immunofluorescent analysis of MILI indicated reduced levels in the SB431542 treated testes compared to DMSO treated controls (Figure 6C). In the third sample MILI was indistinguishable between the SB431542 and DMSO treated samples (Figure 6C). No differences in the levels of FGFR3 were detected between treated and untreated testes (Figure 6C).

Nodal is required for Nanog expression in pre-gastrulation embryos and pluripotent stem cells [52], [60]. We showed that Nodal expression was high in E12.5 and E13.5 male germ cells, but was reduced to 10% of its peak levels by E15.5 (Figure 1). To determine whether blocking Activin/NODAL signaling in E12.5 testes altered expression of Nanog and other pluripotency genes in germ cells we used qRTPCR to examine transcription of Nanog and Sox2, and immunofluorescence to monitor protein levels of SOX2 and OCT4 in E12.5 testes treated with SB431542 or DMSO. After 24 hours of culture, Nanog levels were significantly reduced in SB431542 treated testes compared to DMSO controls (p<0.05), although Sox2 transcription was not affected (Figure 6D). After 72 hours of treatment, both Sox2 and Nanog were downregulated to less than 10% of the levels detected in 24 hour DMSO treated testes (Figure 6D). Despite readily detecting SOX2 and OCT4 protein in the nucleus of E12.5 germ cells, only very low levels of OCT4 and SOX2 were detected in fetal testes treated for 72 hours with SB431542 or DMSO (Figure 6E).

## Discussion

The differentiating testis provides the niche in which the male germ line and spermatogonial stem cells develop. Tight regulation of male germ cell differentiation underpins fertility and prevents the formation of pluripotent germ cell tumours from fetal germ cells. However, the signaling processes that initiate these events and establish this niche are poorly understood.

### TGFβ and Activin control distinct processes during testis and male germ cell development

We demonstrate here that TGFβ and Activin signaling controls distinct processes during testis formation and male germ cell development. Our expression analyses of FACS purified E12.5–E15.5 male and female germ and gonadal somatic cells showed that the genes encoding TGFβ, Activin and NODAL and their receptors are sex, stage and cell type specifically expressed in the developing gonad. Moreover, using well defined ALK4/5/7 and ALK5 inhibitors [47], [48], [49], [50], [51], [52], [53], [54], [55], which completely or partially block SMAD activation (Figure 2A), we demonstrated that TGFβ signaling through ALK5 is required for testis cord formation and to prevent male germ cell entry into meiosis. Using flow cytometric techniques we also showed that Activin (and/or NODAL) signaling promotes Sertoli cell proliferation from as early as E12.5, and that male germ cells do not enter mitotic arrest or differentiate properly when the Activin-NODAL receptors ALK4 and ALK7 are blocked. Therefore, TGFβ and Activin signaling are required for different aspects of testis cord formation and male germ cell development. Consistent with known roles for NODAL in pluripotent stem cells and the early embryo, blocking ALK4/5/7 resulted in reduced male germ cell expression of Nanog, supporting a role for NODAL in germ cell expression of this pluripotency gene. These data define new and essential roles for TGFβ, Activin and NODAL signaling in testis and germ cell development.

We used qRTPCR to analyse expression of Inhba (which encodes ACTIVIN A), Inhbb (which encodes Activin B), Nodal, Tgfb1-3 and their respective type I and type II receptors in male and female somatic and germ cells from E12.5–E15.5 gonads (Figure 1). Tgfb2 and Tgfb3 were expressed at high levels in testicular and ovarian somatic cells, but their expression was not detected in germ cells of either sex. Consistent with a role for TGFβ in somatic cell development, Alk5 and Tgfbr2 were expressed at relatively high levels in E12.5 XY somatic cells (Figure 1), and blocking signaling through ALK5 in E11.5 XY gonads was sufficient to prevent testis cord formation (Figure 2). This phenotype is likely a result of failed Sertoli cell organization, rather than defective specification, as robust levels of SOX9 were detected in the Sertoli cells. TGFβ ligands signal through ALK5, while Activin and NODAL signal through ALK4 and ALK7, thus inhibition of ALK5 in E11.5 male gonads prevented testis cord organization. Similarly, fetal testis cord organization is compromised in Tgfbr3 null mice [46]. Therefore, it is likely that testis cord organization requires TGFβ signaling. Proper cord formation is dependent on endothelial cell migration from the mesonephros [61]. Inhibition of this endothelial cell migration results in disrupted cord formation without affecting Sertoli cell differentiation as shown by SOX9 expression [61]. This phenotype closely resembles the phenotype observed in this study, suggesting that TGFβ signaling might not only regulate testis cord formation by inducing Sertoli cell proliferation at later stages during embryogenesis [39], but also through Sertoli cell proliferation and possibly cell migration at early stages of testis differentiation.

Despite these combined data, mice lacking individual Tgfb genes have normal testis cord formation [44], [45]. However, functional redundancy may obscure testicular phenotypes in these models. Unfortunately, Tgfb double knockouts are embryonic lethal and appropriate compound conditional knockout models are not yet available to examine functional redundancy between these genes. The current ex vivo organ culture model provides an important alternative for demonstrating roles for TGFβs in male sex determination and testis development.

### XY fetal germ cells enter meiosis in the absence of TGFβ signaling

**Figure 7:**
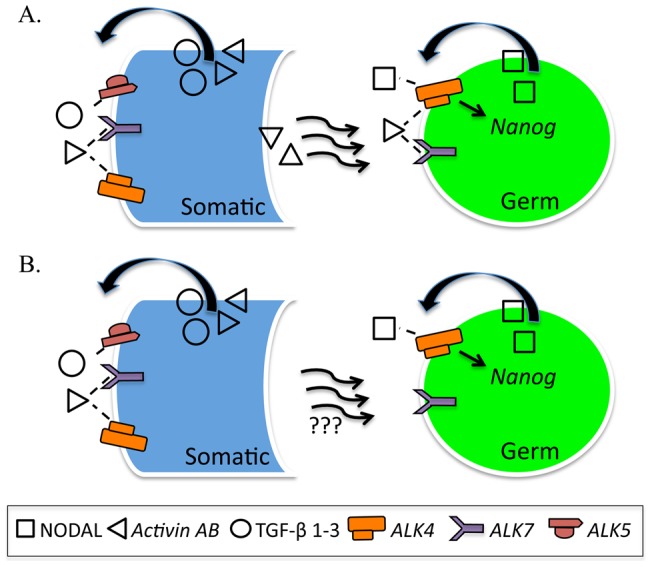
TGFβ/Activin/NODAL signaling in the developing testis. A–B) TGFβ2and TGFβ3 signaling produced from somatic (Sertoli, Leydig or other) cells, regulates testis cord organization through ALK5, while Activin promotes Sertoli cell proliferation through ALK4/7. Male germ cell development and mitotic arrest is promoted primarily by Activin through ALK4/7. (A, B). Activin signaling through ALK4/7 regulates somatic and germ cell development directly (A) or may indirectly lead regulate germ cell differentiation and mitotic arrest by directing somatic cell differentiation and the production of other, unknown ligands by the somatic cells (B). NODAL produced from germ cells positively regulates Nanog expression in germ cells and thereby regulates germ cell pluripotency (A, B). In this model Activin and NODAL have opposing roles in germ cells, promoting and inhibiting differentiation, respectively.

In addition to failed testis cord formation, our data demonstrated that male germ cells aberrantly enter meiosis in ALK5i-I and SB431542 treated E11.5 XY gonads (Figure 4). Similarly, Nodal is specifically expressed by XY germ cells and treatment of E11.5 male gonads with SB431542 results in germ cell entry into meiosis [59]. It was concluded that cell-autonomous NODAL signaling prevents male germ cell entry into meiosis [59]. However, SB431542 blocks ACTIVIN and TGFβ signaling, as well as NODAL signaling, while ALK5i-I only blocks TGFβ signaling through ALK5. Therefore, our study demonstrated that treatment of E11.5 XY gonads with either SB431542 or ALK5i-I blocks testis cord formation, revealing an important role for TGFβ signaling in testis development. Since germ cells are apparently not required for testis cord formation [62], it is unlikely that blocking the action of NODAL accounts for the disrupted testis cord structure observed in our experiments. It is more likely that TGFβ signaling from the somatic cells regulates cord formation in the developing testis. Moreover, blocking ALK5 alone resulted in both disrupted testis cord formation and entry of male germ cells into meiosis, demonstrating that TGFβ signaling is required to prevent germ cells in a fetal testis from entering meiosis. This block of meiosis by TGFβ may be either direct, or indirect. Indirectly, TGFβ may act through signaling from neighboring somatic cells. Hence, blocking TGFβ signaling from somatic cells would lead to both failed testis cord formation and reduced male germ cell differentiation. Consistent with an indirect role, our study demonstrates that multiple TGFβ ligands and their receptors are expressed at high levels in XY somatic cells. Although, Alk5 is expressed in E12.5 male germ cells, the levels were seven fold lower than in somatic cells, and the co-receptor, Tgfbr2, is not expressed by XY germ cells. Therefore, TGFβ is most likely to act indirectly to prevent male germ cells from entering meiosis and depends on Sertoli cell products or organization of the testis cords to facilitate delivery of male germ cell promoting products, rather than direct TGFβ signaling to germ cells (Figure 7).

### Activin promotes XY germ cell differentiation and germ cell mitotic arrest in the fetal testis

Treatment of E12.5 testes with the Activin, TGFβ and NODAL signaling inhibitor (ALK4/5/7 inhibitor) SB431542, resulted in increased germ cell proliferation and decreased Sertoli cell proliferation, while ALK5i-I did not affect germ cell proliferation. Flow cytometric analysis of E12.5 testes treated for 72 hours with SB431542 demonstrated that 14.3% of germ cells were proliferative, while only 4.9% and 3.2% of germ cells were proliferative in ALK5i-I and DMSO treated testes (Figure 5). Using the same assay on E12.5 in vivo isolated testes, around 50% of germ cells are proliferative [27], [28], [56]. Based on the proliferation difference in germ cells of E12.5 testes treated with SB431542 or DMSO for 72 hours, we estimate that around 20–25% of the male germ cell population escaped mitotic arrest in SB431542 treated testes, while essentially all germ cells entered mitotic arrest in ALK5i-I treated testes (compared to DMSO control). As SB431542 blocks ALK4/5/7 and ALK5i-I blocks ALK5, it appears that signaling through ALK4 and/or ALK7 is required for male germ cell mitotic arrest. This conclusion is consistent with the relatively high expression levels of Alk4, and its co-receptors Acvr2a and Acvr2b, but low levels of Alk5 and the absence of its co-receptor Tgfbr2 in male germ cells (Figure 1).

Germ cells entering mitotic arrest upregulate p27^KIP1^ and DPPA4, while losing RB-P and KI67 [27], [28], [56]. However, in the current study immunofluorescent analysis demonstrated reduced p27^KIP1^ and DPPA4, and abnormal maintenance of RB-P and KI67 in germ cells of testes treated with SB431542, but not in DMSO treated testes. Multicolor staining demonstrated that DPPA4 was specifically reduced in KI67 positive germ cells in SB431542 treated samples. Since KI67 expression is normally lost in all fetal male germ cells entering mitotic arrest, this data strongly indicates that the KI67 positive/DPPA4 –ve germ cells detected in the SB431542 treatment have not entered the mitotic arrest or male differentiation programs properly, while other germ cells appear unaffected. It remains unclear what the differences between the different subsets of germ cells are that influence their susceptibility to inhibition of Activin-TGFβ signalling. However, variability in germ cell response to SB431542 treatment in the E12.5 testes may explain the moderate reduction in transcription of Dppa4 in SB431542 treated testes as measured by qRTPCR, while DPPA4 protein levels were substantially reduced only in germ cells that remain KI67 positive (Figure 6B). Despite the variability detected in KI67/DPPA4 expression in SB431542 treated testes, the combined flow cytometric and immunofluorescence data clearly demonstrate that blocking Activin and/or NODAL after male sex determination, but early in testis development, results in compromised male germ cell development.

Analysis of KI67 and DPPA4 staining in SB431542 treated E12.5 testes clearly demonstrated that affected KI67/MVH double positive DPPA4 negative germ cells were mixed with neighbouring unaffected KI67 negative MVH/DPPA4 positive germ cells (Figure 6B). These affected and unaffected germ cells are presumably exposed to similar environments, indicating that they have qualitatively different responses to the treatment. Although perplexing, this stochastic response in germ cells is not unusual, as we have made similar observations in fetal germ cells of Ret null mice (in which all germ cells lack Ret) and in an epigenetic model that we are currently analyzing [63]. Such variation in germ cell development may prove informative by providing important clues to the mechanisms underlying failed germ cell development and formation of germ cell tumours.

### Autocrine Nodal signaling regulates the core pluripotency gene *Nanog* in XY germ cells

The high levels of Nodal and Tgfb1 expression by E12.5–13.5 and E14.5–15.5 male germ cells, respectively (Figure 1), indicate that signaling occurs from male germ cells to either the somatic cells of the developing testis, or to germ cells in autocrine signaling pathways, as suggested by [59]. A possible role of NODAL in this context is the regulation of pluripotency. Nodal has been previously shown to maintain Nanog expression in early embryos and pluripotent cells [52], [60]. Interestingly, when Activin/NODAL signaling was blocked in E12.5 testes levels of Nanog transcription decreased in 24 hours, while Sox2 transcription was unaffected. Moreover, SB431542 also resulted in reduced Nodal and Lefty 1 expression. Considering these findings and the close relationship between fetal germ cells and pluripotent stem cells, an intriguing possibility is that SB431542 blocks autocrine NODAL signaling in the germ cells, resulting in reduced levels of Nodal, Lefty and Nanog transcription. If correct, this indicates that although Activin promotes male germ cell differentiation, NODAL might operate in opposition to this activity. In this capacity NODAL may also play a protective role in the male germ line, preventing male germ cells from entering meiosis [59].

### Activin promotes Sertoli cell proliferation from early stages of testis formation

SB431542 and ALK5i-I treatment had differential effects in the somatic compartment of the testis. Our flow cytometric analysis of E12.5 testes treated with SB431542 or ALK5i-I demonstrated significant reductions in proliferation over the whole the somatic cell population (Somatic cell proliferation: SB431542 12.3±0.81%, DMSO 15.9%±0.6, p = 0.001) and, more specifically, the Sertoli cells (6.3% compared to 33.2%) (Figure 3). Interestingly, Sertoli cell proliferation (9.8% compared to 25.9%) was also decreased by ALK5i-I, but to a lesser extent than for SB431542 treatment. Combined, this data strongly indicates that Activin and TGFβ signaling combine to promote Sertoli cell proliferation during testis formation.

Previous work demonstrated that deletion of Inhba leads to compromised Sertoli-Leydig cell signaling and a later phenotype (ie at E15.5) in Sertoli cell proliferation than observed in this study [39]. Our work extends these findings by demonstrating a requirement for Activin/TGFβ signaling in Sertoli cell proliferation during earlier stages of testis formation. Since inhibiting signaling through ALK5 blocks testis cord formation at E11.5, it appears that TGFβ also contributes to critical cord patterning events that are required for testis formation. Combined, our studies demonstrate that developmental differences occur in TGFβ and Activin/NODAL activity in the somatic and germ cell populations of the developing testis. In this context the lack of sex determination/germ cell phenotypes in single *Tgfb* or *Activin* knockout models indicates that redundancy exists between the Activin and TGFβ signaling networks required for testis formation. Such redundancy would presumably ensure normal testis development and reproductive fitness if one of these pathways were compromised.

### Conclusions

This study identifies new roles for TGFβ and Activin in testis and male germ cell development. We demonstrate that TGFβ signaling regulates Sertoli cell proliferation and formation of testis cords within the developing testis, while Activin (and potentially NODAL) plays key roles in Sertoli cell proliferation, testis cord growth and male germ cell differentiation, including the entry of male germ cells into mitotic arrest. Further delineation of these processes will contribute to understanding the molecular events underlying failed germ cell differentiation in germ cell tumour formation and sub/infertility and will be highly relevant for understanding male reproductive disorders in mammals, including humans.

## Materials and Methods

### Animals

Mice used in all experiments were derived from OG2 (Oct4-GFP; octamer-binding transcription factor 4, also known as Pou5f1) transgenic male (C57BL/6) x CD1 female matings. The presence of a vaginal plug in the morning was used to indicate mating and was recorded as E0.5. All animal procedures were carried out under Murdoch Childrens Research Institute Animal Ethics Committee or Monash University Animal Ethics Committee approvals.

### Fluorescent Activated Cell Sorted Somatic and Germ Cell RNAs and qRTPCR

Germ and somatic cells were previously sorted from Oct4-GFP embryonic gonads and RNA extracted, and amplified as described [27], [64]. These amplified RNA libraries provide germ and somatic cell specific sources of RNA for gene expression analyses in E12.5-E15.5 testes and ovaries. The purity of this material was extensively verified using independent assays [27], [64]. For example, flow cytometric analysis indicated that these samples represent somatic and germ cell populations of ≥99% purity [64], [65]. Details of primer and UPL Probe sets used are shown in Table 1. Results were analysed using LightCycler 480 software (Roche) and relative expression levels were determined by normalisation against verified reference genes Canx and Mapk1 [64]. Each sampling point (E12.5–E15.5) and cell type (germ and somatic cells) was analysed in biological triplicate and PCRs performed in technical duplicate.

**Table 1:** Primers for qRTPCR analysis.

Organism	Gene	Genbank/ENSEMBL	UPL probe	Primer name	Sequence
mouse	Acvr1b	NM 007395.3	49	left	tgcttgagctttctgtgcat
mouse	Acvr1b	NM 007395.3	49	right	gagaagcagcagcactcaga
mouse	Acvr1c	NM 001033369.1	72	left	tggtaacagaagatcacatcagtg
mouse	Acvr1c	NM 001033369.1	72	right	catgcatggtccctgttaaa
mouse	Acvr2a	NM 007396.3	95	left	ttgactttcctcccaaagaatc
mouse	Acvr2a	NM 007396.3	95	right	ttccttagcttagcagctcca
mouse	Acvr2b	NM 007397.2	32	left	gacctccgtcaccaatgtg
mouse	Acvr2b	NM 007397.2	32	right	ctacgtgtcccgggcttag
mouse	Canx	NM 007597.2	50	left	ccacataggaggtctgacagc
mouse	Canx	NM 007597.2	50	right	caccaccagcattccttaaaa
mouse	Inhba	NM 008380	106	left	attggctggaatgactggat
mouse	Inhba	NM 008380	106	right	ggcactccccctcacaata
mouse	Inhbb	NM 008381.3	81	left	agcacttgcaggtctacgtgt
mouse	Inhbb	NM 008381.3	81	right	acagccacgtcaagctctc
mouse	Mapk1	NM 01194	21	left	gcccttcagagcactcca
mouse	Mapk1	NM 01194	21	right	aacaccaaaaaggcatccac
mouse	Nodal	NM 013611.3	52	left	gagggcgagtgtcctaacc
mouse	Nodal	NM 013611.3	52	right	tctggatgtaggcatggttg
mouse	Tgfb1	NM 011577.1	66	left	caattcctggcgttaccttg
mouse	Tgfb1	NM 011577.1	66	right	agacagccactcaggcgtat
mouse	Tgfb2	NM 009367.3	45	left	ggactaagcaagtcttctgtgga
mouse	Tgfb2	NM 009367.3	45	right	gaagcttcggcagacacg
mouse	Tgfb3	NM 009368.3	38	left	taccctagaccccgttgct
mouse	Tgfb3	NM 009368.3	38	right	aggctccccggatacttg
mouse	Tgfbr1	NM 009370.2	67	left	agtcagtccgttgggtcttc
mouse	Tgfbr1	NM 009370.2	67	right	gtaaaacccaggctcaacca
mouse	Tgfbr2	NM 009371.3	75	left	caacaccagtgggttccatt
mouse	Tgfbr2	NM 009371.3	75	right	gtgcgccattcaaatcct
mouse	Lefty1	NM_010094	Sybr	left	tgccattgttcccttaatttg
mouse	Lefty1	NM_010094	Sybr	right	tggcttaggtccagagtcaag
mouse	Dppa4	NM_028610	Sybr	left	gctgaggtcagaggtcttttg
mouse	Dppa4	NM_028610	Sybr	right	gctcttctgcgctccact
mouse	Dnmt3l	BC083147	Sybr	left	agtcagaagcaggagcaagc
mouse	Dnmt3l	BC083147	Sybr	right	agcgggagaaggcagttc
mouse	Nanog	NM_028016	Sybr	left	ttggagacagtgaggtgcat
mouse	Nanog	NM_028016	Sybr	right	ctggggtaagggtgttcaag
mouse	Sox2	NM_011443	Sybr	left	cgcccagtagactgcaca
mouse	Sox2	NM_011443	Sybr	right	ccctcacatgtgcgacag

### Organ Culture

E11.5 gonad and mesonephros of E12.5 gonad only samples were collected from Oct4-GFP transgenic embryos and sexed using PCR as previously described [66]. XX and XY gonads were placed onto 25 mm Polycarbonate, polyvinyl pryolidine free (PVPF), 12.0 micron filters (GE Water and Process Technologies) in single well organ culture dishes (NUNC) containing 1500 μl organ culture media (250 μM sodium pyruvate, 2 mM L-glutamate, 1X non-essential amino acids, 1 mM N-acetylcysteine, 55 μM β-mercaptoethanol, FCS (10%)) in DMEM in the inner well and 3 ml of PBS in the outer well to maintain a humid environment. 10 μM (final concentration) ALK4/5/7 inhibitor (SB431542, Sigma S4317, stock dissolved in DMSO), 2.3 μM ALK5i-I (TGFβ RI Kinase Inhibitor I, Calbiochem, Calbio 616451, stock dissolved in DMSO) or DMSO (1 in 1000), were added to the culture medium at the outset of the experiment. The inhibitor concentrations used were based on previous analyses of their efficacy in blocking Activin/TGFβ signaling through ALK4/5/7 and ALK5 respectively [47], [48], [49], [50], [51], [52], [53], [54], [55]. Gonads were cultured for 24, 72 or 96 hours in 5.0% CO2 at 37°C with organ culture media changed daily. All organ cultures were replicated at least 3 times using 4–8 individual gonads per replicate. EdU was added at a final concentration of 20 μM to the organ culture media for the final 2 hours of culture. Gonads were removed from the filters, photographed under bright field and fluorescence optics, dissociated using trypsin and fixed for flow cytometric analysis, immunofluoresence or used for RNA or protein preparation. Flow cytometry was performed as described [27], [28], [56]. Statistical significance was determined using the two-tailed t test assuming equal variance. A p value of less than 0.05 was considered significant.

### Tissue fixation and immunofluorescence

Cultured gonads were fixed in PBS containing 4% paraformaldehyde for 1–3 hours at RT or overnight at 4°C and mounted in optimal cutting temperature. Cryosections were cut at 10 μm, and immunofluorescence performed as previously described [27], [28], [56]. Primary and secondary antibody details are provided in Tables 1 and 2, respectively. Where a combination of rabbit antibodies was used to stain tissues, the first antibody was bound and detected using an Alexa 594 anti-rabbit secondary antibody and then the second primary antibody applied after direct labelling using the Zenon 488 Labelling Kit (Life Technologies) according to manufacturer's instructions. Staining of treated samples and their corresponding controls was performed in the same experiment, with each slide receiving antibody dilutions from the same batch prepared stock. Staining with each marker was repeated as a minimum in biological triplicate. TUNEL staining was performed as described in Miles et al 2012 [63].

**Table 2:** Antibodies for immunofluorescence (IF) and western blotting.

Protein	Source & Catalogue #	Species	Dilution
FOXL2	Dr Dagmar Wilhelm	Rabbit	IF: 1:1000
γH2A.X (Ser139) (20E3)	Cell Signaling 9718	Rabbit	IF: 1:100
MVH	Abcam 13840–100	Rabbit	IF: 1:10000
SOX9	Dr Dagmar Wilhelm	Rabbit	IF: 1:200
SOX2	AbCam 97959	Rabbit	IF 1:1000
OCT4	Cell Signalling 2840	Rabbit	IF 1:500
MILI	Cell Signalling 2071	Rabbit	IF 1:200
SMAD2	Cell Signalling 5339	Rabbit	W 1:1000
P-SMAD2	Cell Signalling 3108	Rabbit	W 1:1000
RB-P	Cell Signalling 9308	Rabbit	IF 1:1000
p27^KIP1^	Cell Signalling 3688	Rabbit	IF 1:200
FGFR3	Santa Cruz sc123	Rabbit	IF 1:200
KI67	NeoMarkers Rm-9106	Rabbit	IF 1:50
DPPA4	R&D Systems AF3730	Goat	IF 1:400
SYCP3	Abcam 15902	Rabbit	IF 1:500
STRA8	Abcam 49405	Rabbit	IF 1:1000
Cleaved-PARP	Cell Signalling 9544	Rabbit	IF 1:100

### RNA isolation and qRTPCR

Total RNA was isolated from 6–8 testes treated with DMSO or SB431542 for 24 or 72 hours using standard Trizol (Life Technologies) extraction method. The RNA was DNase treated and quantified using a Nanodrop spectrometer. Reverse transcription qRTPCR was performed as previously described [64] using primers listed in Table 1 and sybr green quantification. Samples were examined in biological triplicate and technical duplicate.

### Immunoblotting

Western blot analyses were performed using samples prepared as previously described [27], [56]. Two gonads were boiled in 15 µl of 2x Laemmli buffer and protein separated on 10% pre-cast NuPage Tris-Acetate gels (Life Technologies) and blotting performed using standard procedures. Antibodies are listed in Table 2.
